# A Case of Chronic Q Fever Treated With Doxycycline and Trimethoprim–Sulfamethoxazole With a Favorable Outcome

**DOI:** 10.1155/crdi/9811461

**Published:** 2025-09-23

**Authors:** Pairie M., Dutta N., Erayil S. E., Van't Hof J. R., Paim A. C.

**Affiliations:** ^1^Division of Internal Medicine, M Health Fairview University of Minnesota Medical Center East Bank, Minneapolis, Minnesota, USA; ^2^Division of Infectious Diseases and International Medicine, M Health Fairview University of Minnesota Medical Center East Bank, Minneapolis, Minnesota, USA; ^3^Division of Cardiovascular Diseases, M Health Fairview University of Minnesota Medical Center East Bank, Minneapolis, Minnesota, USA

**Keywords:** chronic Q fever treatment, Coxiella, *Coxiella burnetii*, Q fever

## Abstract

In United States, Q fever cases increased from < 50 to 200 from 2000–2019. Case: 31-year-old female with fever after exposure to unpasteurized milk. Investigation revealed positive Q fever IgG. Trimethoprim–sulfamethoxazole and doxycycline were started due to QTc prolongation. Evidence is limited on treatment without hydroxychloroquine, and the use constraints might prompt additional studies.

## 1. Introduction

Q fever is a zoonotic condition caused by *Coxiella burnetii*, a Gram-negative, intracellular bacterium. It was first described in Queensland, Australia, in 1935. As the cause could not initially be identified, the term “Q (query) fever” was proposed in 1937 [1]. The illness is now known to be associated with livestock and farm workers handling ungulates [2].

In the United States, the condition became nationally notifiable in 1999. Since then, barring some oscillations from 2008 to 2012, the number of cases is gradually increasing from less than 50 in the year 2000 to approximately 200 in 2019. Most cases of Q fever are acute in nature, while some may become chronic. The principal route of transmission is by inhalation of aerosol particles containing the bacterium. Less often, the contamination can be from consumption of unpasteurized dairy products [3, 4]. Primary reservoirs are ruminants including sheep, goats, and cattle [3]. The clinical syndrome can be acute or chronic. The former comprises two typical presentations: atypical pneumonia and hepatitis. It has been theorized that the spectrum and the quality of such clinical categories is potentially related to the route in which the infection was transmitted [1, 5]. Almost invariably transient bacteremia will take place with subsequent involvement of other organs and, in specific hosts, culminating in the chronic form of disease with endocardial and vascular involvement.

Treatment for acute Q fever is typically a two-week course of doxycycline, while the recommended treatment for chronic Q fever is 18–24 months of doxycycline plus hydroxychloroquine [4, 6, 7].

## 2. Presentation

A 31-year-old female presented to the emergency department with shortness of breath, generalized weakness, and intermittent fever. She has a history of Marfan syndrome, with biosynthetic mitral valve replacement (MVR) 16 years prior to initial presentation.

She had spent the previous year outside of the United States doing traveling, including Ethiopia, Somalia, and Kenya. Of note, she drank unpasteurized camel milk. She was feeling well until 3 months before her presentation to our hospital. At that time, she was hospitalized in Kenya for pneumonia and was treated with antibiotics. Following that time, she had felt consistently weak. She was hospitalized again in Kenya approximately 2 weeks before presenting to our institution with hypotension and fever and was treated with antibiotics. She was discharged on cefixime and linezolid for a presumable infection. Unfortunately, attempts to reach the hospital in Kenya were unsuccessful.

She was discharged from the hospital in Kenya, returned to the United States, and was admitted to our institution the following day. On admission, she had a temperature of 98.3 F, heart rate of 85 beats per minute, blood pressure of 99/65 mmHg, respiratory rate of 20 breaths per minute, and oxygen saturation of 96%. Laboratory tests were significant for hemoglobin of 8.9 g/dL (normal range: 11.7–15.7 g/dL), alkaline phosphatase of 110 U/L (normal range: 35–105 U/L), AST of 39 U/L (normal range: 10–35 U/L), CRP of 39.31 mg/L (normal range < 5 mg/L), procalcitonin 0.26 ng/mL (normal range < 0.05 ng/mL), and N-terminal Pro BNP: 2983 pg/mL (normal range: 0–450 pg/mL). The patient had extreme fatigue but was oriented and able to answer questions. Lung exam was unremarkable. No murmur was noticed on cardiac exam. No skin lesions or lymphadenopathy were noted. Given preceding fever and presence of risk factors for endocarditis, she was initially started on empiric vancomycin and meropenem after collection of blood cultures on the day of admission. HIV serology, QuantiFERON-TB Gold, bacterial blood cultures, MRSA PCR from nasal swab, malaria/parasite blood smear (3 samples), hepatitis A, and C serologies were all negative. Hepatitis B serology indicated immunity. Antibiotics were subsequently narrowed to vancomycin and ceftriaxone on the second day of hospitalization. Transthoracic and transesophageal echocardiograms were obtained and did not show evidence of cardiac vegetation. At that point, given the report of consumption of unpasteurized milk, we decided to obtain *C. burnetii* and Brucella serology as well as Karius (next generation sequencing) from blood.

Six days into her admission, she was still febrile and developed a more pronounced elevation of temperature at 102.4°F. On that date, antibiotics were changed to vancomycin and piperacillin/tazobactam. Two days later (eighth day of hospitalization), Karius testing results came back highly positive for *C. burnetii* (29,908 molecules per mL), and *C. burnetii* serology resulted with elevated titer of Phases I and II IgG antibody (1: 65,536 for both). Doxycycline was therefore started, and piperacillin/tazobactam was discontinued on that same day. There was resolution of fever 48 h later (10 days into hospital stay).

The presence of high titer of Phase I IgG antibody and the pre-existing history of cardiovascular genetic disorder prompted us to consider adjustment of treatment for chronic Q fever. According to the recommendations from CDC and Dutch consensus guidelines, hydroxychloroquine would need to be added to doxycycline. The main constraint to initiation of the medication was a baseline prolonged QTc of 519. We consulted cardiology, and it was concluded that the risk of arrhythmias would outweigh the benefits of hydroxychloroquine. Trimethoprim–sulfamethoxazole (TMP-SMX) was added to doxycycline, allowing for two agents to be utilized against the infection. A PET scan ruled out cardiovascular involvement.

**Figure 1:**
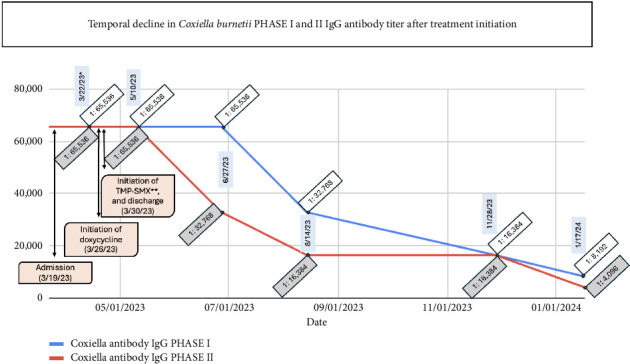
^∗^Initial serology test was collected on 3/22/23; however, the result was not available until 3/26/23. ^∗∗^TMP-SMX: Trimethoprim–sulfamethoxazole.

The patient was dismissed after 11 days of hospitalization with resolution of the fever and improvement of her fatigue. Follow-up in infectious disease clinic showed that symptoms continued to be in remission. Her Q fever serology titers have been progressively declining as seen on Figure 1.

## 3. Discussion

We presume that the most likely cause of disease acquisition was ingestion of the organism in unpasteurized camel milk. The patient's initial presentation with pneumonia at the hospital in Kenya could potentially have been the acute phase of the disease. If this hypothesis is true, the subsequent months harboring the infection could have been responsible for the high titer of IgG to Phase I. It was observed that the titer of IgG to Phase II was similarly elevated. Even though this is a marker of acute infection, in some patients, it can be elevated for up to 1 year.

It is intriguing that the patient, although with pre-existing cardiac condition, did not have evidence of endocardial or vascular disease. Per literature [1, 4], chronic Q fever is more firmly defined by clinical symptoms lasting longer than 6 months (up to decades) from initial exposure. If our presumption is accurate and the patient acquired the disease 3 months before the hospital presentation, she could be in an evolving chronic phase and establishment of serologic diagnosis potentially occurred before cardiovascular disease could take place. One argument we can use in that favor is the persistence of acute phase antibodies at the time of diagnosis.

In regards of therapeutic options for chronic fever, doxycycline is recommended in addition to hydroxychloroquine. The latter is a weak lysosomotropic base which was demonstrated to inhibit *C. burnetii* intracellular growth in the phagolysosome by changing the pH [8, 9]. On the other hand, a more recent publication [10] has been questioning the in vivo therapeutic effect of hydroxychloroquine. Additionally, some studies have explored the combination of doxycycline plus TMP-SMX or other antimicrobial agents with potentially favorable responses [11, 12]. We do acknowledge the limited evidence on treatment with doxycycline in combination with TMP-SMX; however, we anticipate that the constraints related to the use of hydroxychloroquine will prompt clinicians and researchers to study the efficacy of alternative combinations for treatment of chronic Q fever in detail.

## Data Availability

The research data are not shared.
